# ADAM15 Is Functionally Associated with the Metastatic Progression of Human Bladder Cancer

**DOI:** 10.1371/journal.pone.0150138

**Published:** 2016-03-01

**Authors:** Guadalupe Lorenzatti Hiles, Amanda Bucheit, John R. Rubin, Alexandra Hayward, Angelica L. Cates, Kathleen C. Day, Layla El-Sawy, L. Priya Kunju, Stephanie Daignault, Cheryl T. Lee, Monica Liebert, Maha Hussain, Mark L. Day

**Affiliations:** 1 Division of Urologic Oncology, Department of Urology, University of Michigan, Ann Arbor, Michigan, United States of America; 2 Comprehensive Cancer Center, University of Michigan, Ann Arbor, Michigan, United States of America; 3 Translational Oncology Program, University of Michigan, Ann Arbor, Michigan, United States of America; 4 Hematology/Oncology, Department of Internal Medicine, University of Michigan, Ann Arbor, Michigan, United States of America; 5 School of Public Health, University of Michigan, Ann Arbor, Michigan, United States of America; 6 European Egyptian Pharmaceuticals, Alexandria, Egypt; 7 Department of Pathology, University of Michigan, Ann Arbor, Michigan, United States of America; University of Kentucky College of Medicine, UNITED STATES

## Abstract

ADAM15 is a member of a family of catalytically active disintegrin membrane metalloproteinases that function as molecular signaling switches, shed membrane bound growth factors and/or cleave and inactivate cell adhesion molecules. Aberrant metalloproteinase function of ADAM15 may contribute to tumor progression through the release of growth factors or disruption of cell adhesion. In this study, we utilized human bladder cancer tissues and cell lines to evaluate the expression and function of ADAM15 in the progression of human bladder cancer. Examination of genome and transcriptome databases revealed that ADAM15 ranked in the top 5% of amplified genes and its mRNA was significantly overexpressed in invasive and metastatic bladder cancer compared to noninvasive disease. Immunostaining of a bladder tumor tissue array designed to evaluate disease progression revealed increased ADAM15 immunoreactivity associated with increasing cancer stage and exhibited significantly stronger staining in metastatic samples. About half of the invasive tumors and the majority of the metastatic cases exhibited high ADAM15 staining index, while all low grade and noninvasive cases exhibited negative or low staining. The knockdown of ADAM15 mRNA expression significantly inhibited bladder tumor cell migration and reduced the invasive capacity of bladder tumor cells through Matrigel^TM^ and monolayers of vascular endothelium. The knockdown of ADAM15 in a human xenograft model of bladder cancer inhibited tumor growth by 45% compared to controls. Structural modeling of the catalytic domain led to the design of a novel ADAM15-specific sulfonamide inhibitor that demonstrated bioactivity and significantly reduced the viability of bladder cancer cells *in vitro* and in human bladder cancer xenografts. Taken together, the results revealed an undescribed role of ADAM15 in the invasion of human bladder cancer and suggested that the ADAM15 catalytic domain may represent a viable therapeutic target in patients with advanced disease.

## Introduction

Bladder cancer is the fourth most common cause of cancer, and the eighth most common cause of cancer death in men. An estimated 74,000 men and women will be diagnosed and 16,000 people will die of bladder cancer by the end of 2015 [1]. While the majority of bladder cancers present as noninvasive early stage tumors, up to one third of non-muscle invasive disease will progress to muscle invasive disease and metastasize over time [2]. Despite the effectiveness of cisplatin-based chemotherapy for locally advanced and metastatic bladder cancer, the lack of durability of responses and the absence of second-line therapy point to the need for more effective treatments [3, 4]. Critical to new therapeutic developments are the identification of specific oncogenic pathways with promising therapeutic interventions, and the proper identification of patients who are likely to benefit from a given therapy (individualized cancer therapy).

Most bladder cancers express elevated levels of the epidermal growth factor receptor (EGFR) [5,6,7]. EGFR modulates cell growth and proliferation as well as regulating gene expression, angiogenesis, motility and apoptosis resulting in increased malignant potential [8,9,10]. Elevated EGFR is common in primary bladder cancers with about 50% of cases exhibiting overexpression [8]. Additionally, a majority of metastatic bladder tumors have been shown to express EGFR [11]. Further, EGFR overexpression appears to be a significant negative predictor of survival [5]. Thus, biological pathways that enhance growth factor signaling through EGFR are likely to contribute to bladder cancer progression and metastasis. Cell-bound EGFR ligands, such as heparin-binding epidermal growth factor (HB-EGF), amphiregulin, transforming growth factor alpha (TGFα), and betacellulin are known to be released through the action of membrane-bound proteases including members of the ADAM (A Disintegrin And Metalloproteinase) family [12–14].

The ADAM family consists of approximately 40 proteins, but only a few exhibit proteolytic activity [15–17]. The proteolytically active ADAMs, or sheddases, traffic to the membrane in a latent form which can be activated by the proteolytic shedding of an N-terminal inhibitory prodomain [12,13]. The enzymatically active protein cleaves and releases several physiologic cell surface proteins including membrane anchored growth factors and their receptors, ecto-enzymes, and cell adhesion molecules such as E-cadherin and N-cadherin. These functions place specific ADAMs in fundamental roles for cellular signaling and the regulation of cell adhesion and cellular motility [12,15]. Several ADAMs have also been implicated in human tumorigenesis [13,14,16,18,19] and increased expression and function of these ADAMs often correlate with tumor progression and aggressiveness of disease [18,20].

One of the catalytically active ADAMs, ADAM15, has been reported to be overexpressed in numerous malignancies including melanoma, prostate cancer and breast cancer [20,21]. The list of substrates for ADAM15 includes several key cell regulatory molecules including E-cadherin and N-cadherin, desmoglein and the EGFR ligands, Transforming Growth Factor Beta (TGFβ), amphiregulin, epiregulin and HB-EGF [16,21,22]. The level of overexpression of ADAM15 in breast and prostate cancer has been correlated with tumor aggressiveness and metastatic progression [20]. We have shown that the inhibition of ADAM15 expression in PC-3 prostate cancer cells reduced tumor growth and prevented metastasis [23]. A link between ADAM15 and angiogenesis has also been noted [24,25].

The biological and molecular profiles of bladder cancers suggested that overexpression and activation of ADAM15 may be relevant to the progression of this disease. First, bladder cancers express high levels of EGFR, where local release of EGFR ligands would promote bladder cancer growth [5–7,11]. Second, E-cadherin, another substrate for ADAM15, has been found in the enzymatically processed form in both serum and urine samples from bladder cancer patients which also correlated with poor clinical outcome [26,27]. Third, bladder cancer progression is closely linked to angiogenesis [9,28–30], which may also be affected by the biological activity of ADAM15 [24,25].

ADAM15 is emerging as a potential regulator of the tumor microenvironment and its promise for therapeutic targeting is rising. Preliminary examination of ADAM15 expression in tissue, cell lines and xenograft models of human cancer (histologically similar to the primary tumor) suggested its functional role in the progression of human bladder cancer. However, little was known about the expression levels of ADAM15 in human bladder cancer or how ADAM15 might functionally mediate the invasion and metastasis of bladder cancer cells. In the current study, we set out to validate the association of ADAM15 expression with advanced human bladder cancer and establish the functional participation of ADAM15 in the progression of this disease.

## Materials and Methods

### Cell Culture

Human bladder cancer cell lines UM-UC-6 [31,32] and UM-UC-9 [32] were obtained from the originator (H.B. Grossman, The University of Texas-MD Anderson Cancer Center, Houston TX). SV-40 immortalized Human Uroepithelial Cells (SV-HUC-1) [33] were a kind gift from C.A. Reznikoft (University of Wisconsin, Madison, WI). Established Human Umbilical Vein Endothelial Cells (HUV-EC-C) were acquired from the American Type Culture Collection (ATCC, Manassas, VA). Cells were utilized within short-term passage and tested by restriction fragment length polymorphism (RFLP) for authentication (Research Animal Diagnostic Laboratory, Columbia, MO). Cells were maintained in Dulbecco’s Modified Eagle Medium (DMEM; Life Technologies, Grand Island, NY), with high glucose, supplemented with 10% heat-inactivated fetal bovine serum (FBS) and 1% penicillin-streptomycin (Life Technologies). The HUV-EC-C cells were maintained in Endothelial Cell Growth Medium EBM-2^TM^ (Lonza Inc., Allendale, NJ) supplemented with EGM™-2 BulletKit™ (Lonza Inc.) and 10% FBS.

### Generation of ADAM15 Knockdown Cell Lines

UM-UC-6 and UM-UC-9 ADAM15-knockdown bladder cancer cells were generated using a lentivirus carrying ADAM15-specific knockdown short hairpin RNA (shRNA) (shA15), or empty vector control sequence and/or scrambled shRNA sequence designed as a control for off-target effects (ctlA15) as we previously reported [22,23]. The forward and complementary target sequences for ADAM15 were 5’-AACCCAGCTGTCACCCTCGAA-3’ and 5’-TTCGAGGGTGACAGCTGGGTT-3’.

### Patient Samples

The tumor samples for this work were obtained preserving patient’s confidentiality under written informed consent at the University of Michigan Bladder Cancer Tissue Bank. Each source complies with the Medical School Institutional Review Board approved protocol (IRBMED HUM00042401). The tissue microarray consisted of 110 cores on 37 bladder cancer patients, and included samples from normal bladder as well as superficial, invasive and metastatic bladder cancers.

### Protein Isolation and Immunoblotting

Cells were harvested by scraping and the resulting cell pellet was washed twice with phosphate-buffered saline (PBS), and frozen at -80°C prior to extraction. Cell pellets were lysed in lysis buffer (50 mM Tris [pH 7.6], 120 mM NaCl, 0.5% NP40, 1 mM EGTA, 100 μg/mL phenylmethysulfonyl fluoride, 50 μg/mL aprotinin). Extracts were cleared by centrifugation at 10,000 RPM for 10 minutes, and supernatants were collected and protein quantitated using the Bradford protein assay (Bio-Rad, Hercules, CA). Cellular extracts (20 μg/lane) were then separated on pre-cast gradient 4–20% Tris-glycine SDS-polyacrylamide gels (Novex, Life Technologies, Carlsbad, CA) and transferred to a reinforced 0.2 μm nitrocellulose membrane (EMD Millipore, Billerica, MA). Membranes were then blocked, probed, and developed. Blocking buffer consisted of 10% nonfat dry milk in TBST (Tris buffered saline with 0.1% Tween 20; Sigma-Aldrich Co., Saint Louis, MO). Primary and secondary antibody incubations were performed in 2.5% nonfat dry milk in TBST. Primary antibodies against GAPDH (mouse monoclonal MAB374 antibody, lot 2322571, EMD Millipore, Billerica, MA) and ADAM15 (rabbit polyclonal AB19036 antibody, Lot 2316790, EMD Millipore) were utilized at final dilution 1:10,000 and 1:2,000, respectively. The secondary antibodies were goat anti-mouse IRDye^TM^ 680RD 1:5,000 (926–68070, lot C30502-01, Li-Cor, Lincoln, NE) and goat anti-rabbit IRDye^TM^ 800CW 1:10,000 (C30521-01, lot C30521-01, Li-Cor). Membranes were imaged with the Li-Cor Odyssey CLX^TM^ (Li-Cor, Lincoln, NE) following the manufacturer’s instructions.

### Immunohistochemistry

Paraffin embedded tissue sections were deparaffinized and treated with peroxide in methanol to block endogenous peroxide activity. Antigen retrieval was performed using citrate buffer antigen retrieval following the manufacturer’s procedure (Citra, Biogenex, San Ramon, CA). Immunostaining was performed using the avidin-biotin complex staining (ABC; Vector Laboratories, Burlingame, CA). Primary rabbit polyclonal antibody to ADAM15 (AB19035, lot 0603024760, EMD Millipore) was diluted 1:50 in 5% FBS in PBS. A pre-diluted rabbit isotype solution (Life Technologies, Carlsbad, CA) was used as a negative control. The reaction was completed by development for 5 minutes with substrate (3,3’-diaminobenzidine plus peroxide). The slides were then counterstained with hematoxylin. Slides were evaluated by a pathologist (LPK) and graded for staining intensity (on a 1–4 scale; 1 = negative, 2 = weak, 3 = intermediate, 4 = strong) and percentage of tumor cells staining. An “intensity score” (staining intensity multiplied by percentage of cells staining) was used to compare the results.

### Wound Healing Assay

UM-UC-9 and UM-UC-6 cells were seeded into 6-well tissue culture dishes and allowed to reach confluence. The monolayer of cells was scratched with a new 5 mL pipette tip across the center of the wells to form a cross. After scratching, the wells were gently washed twice with medium to remove the detached cells and then replenished with fresh DMEM supplemented with 10% FBS. Photographs were taken and digitalized to document the initial fissure. UM-UC-9 and UM-UC-6 cells were then incubated for 18–20 and 12 hours, respectively. Photographs were taken and digitalized to document the migration. The healing gap was measured by Image Analysis Software^TM^ from Olympus (Olympus Corporation, Center Valley, PA).

### Matrigel^TM^ Invasion Chamber Assay

BD BioCoat^TM^ Matrigel^TM^ Invasion Chambers (BD Biosciences, Bedford, MA) were utilized. The 24-well chambers were rehydrated with PBS and ctlA15 and sh15 UM-UC-9 or UM-UC-6 cells were seeded onto the chambers, according to the manufacturer’s procedure (BD Biosciences). A volume of 0.2 mL of cell suspension containing 1x10^5^ cells/mL in serum free medium was added to each insert. A volume of 0.5 mL of DMEM plus 10% FBS was added to the inferior well. Chambers were incubated at 37°C, 5% CO_2_ atmosphere and UM-UC-9 and UM-UC-6 cells were allowed to migrate against a serum gradient for a period of 24 or 12 hours respectively. After incubation, non-invading cells were removed from the upper surface of the insert membrane with cotton tipped applicators. The cells on the lower surface of the membrane were fixed with 10% formalin and stained with crystal violet (BD Bioscience). Cell counting was performed from photomicrographs of 3–5 fields per insert. Each of the cell lines were assayed at least three times.

### Transendothelial Migration Assay

BD BioCoat^TM^ inserts and 24-well plates (BD Biosciences) were utilized for transendothelial migration assay. Fibronectin was added to coat each insert (Sigma-Aldrich Co.) at a concentration of 50 μg/mL. HUV-EC-C endothelial cells were then seeded onto the insert at a concentration of 1x10^5^ cells/250 μL EBM-2 medium plus supplements. HUV-EC-C cells were incubated for 48 hours to form a monolayer. UM-UC-9 and UM-UC-6, ctlA15 and shA15, cells were maintained for 24 hours and seeded onto the invasion inserts at a density of 1x10^5^ cells/250 μL in serum free medium. Control inserts were loaded with medium to assess the migratory background of the endothelial cells. The lower chamber was filled with 300 μL of EBM-2 plus 10% FBS. The chambers were then incubated to allow the tumor cells to migrate through the endothelial layer toward a serum gradient. UM-UC-9 cells were incubated for 24 and UM-UC-6 for a period of 12 hours. Non-transmigratory cells were removed with a cotton swab. Next, the migratory tumor cells were fixed with 10% formalin and stained with crystal violet and photographed to quantify the tumor cell migration. Three fields per insert were photographed and cells counted with assistance of ImageJ [34]. Each condition was run in triplicate.

### Structure-based Design of a Novel Chemical Inhibitor Targeting the Catalytic Domain of ADAM15

The catalytic site of ADAM15 is unique and suggests the possibility of designing novel chemical probes that are selective for the ADAM15 metalloproteinase activity. This approach led to the initial identification and validation of the non-specific inhibitor, marimastat, as a structural probe with excellent predicted binding and inhibitory potential against the active site of ADAM15. Further structural modeling led to the identification of the biphenyl sulfonamide metalloproteinase inhibitor, PD166793 [35], which is endowed with several properties that make it an attractive starting point for analog synthesis of ADAM15 probes. Preliminary structure-based design, taking into account the unique amphipathic properties of the ADAM15 S1’ pocket, allowed us to design and synthetize a new lead compound, adamastat, which is designed as a selective probe for ADAM15. Optimal binding modes and relative binding energies of our ADAM15 metalloproteinase inhibitor was calculated using the widely accepted AutoDock Vina program for molecular docking and virtual screening [36] as represented in S1 Table. These calculations, predict that adamastat should bind selectively and with high affinity to the catalytic site of ADAM15.

### Fluorescence Resonance Energy Transfer (FRET) Assay

The activity of recombinant ADAM15 catalytic domain (A15cat) was determined using FRET based assays which measure the cleavage of a fluorogenic ADAM15 substrate peptide. Inhibition of the catalytic activity of A15cat by marimastat, PD166793 and adamastat was determined using a fluorogenic peptide (dabcyl-HGDQMAQKSK(5FAM-NH2) derived from the ADAM15 cleavage site in the low affinity IgE receptor (CD23) [37]. In these experiments the substrate at a concentration of 1 μM was incubated with recombinant A15cat (0.5 μg/mL) for 24 hours at 24°C in 384 well plates (Corning, Corning, NY) with inhibitor in phosphate buffered saline solutions. Fluorescence was measured using a PHERAstar (BMG LABTECH Inc., Cary, NC) fluorescence plate reader (excitation at 485 nm and emission at 530 nm).

### Cell Proliferation Analysis

To study the effect of our ADAM15 knockdowns on cell proliferation, ctlA15 and shA15 UM-UC-9 and UM-UC-6, cells were seeded into 96-well plates at a density of 1,000 and 500 cells/well respectively in 200 μL of medium. End points were assessed after 12, 24, 48 and 72 hours of incubation.

In a similar fashion, the anti-proliferative effect of adamastat was analyzed by plating SV-HUC-1, UM-UC-9 and UM-UC-6 cells at a concentration of 2,000, 1,000 and 500 cells/well respectively in 100 μL of medium. Cells were allowed to attach overnight and treated with 100 μL/well of a 2X adamastat solution (at 5 μM final concentration) or equivalent concentration of DMSO as vehicle control. Consequently, cells were placed in incubator for 12, 24, 48, 72, 96 and 120 hours.

Cell proliferation was assessed at each time point through bioreduction of a water soluble MTS tetrazolium (3-(4,5-dimethylthiazol-2-yl)-5-(3-carboxymethoxyphenyl)-2-(4-sulfophenyl)-2H-tetrazolium) salt to its formazan product by the metabolically active cells [38]. After addition of 40 μL of MTS/phenazine methosulfate (PMS) (20:1) solution (Promega, Madison, WI), the cells were incubated for 2 hours at 37°C and the absorbance of the formazan product was measured at 490 nm.

### Cell Viability Analysis

SV-HUC-1, UM-UC-9 and UM-UC-6 cells were seeded into 96-well plates at a concentration of 2,000, 1,000 or 500 cells/well respectively in 100 μL of DMEM plus 10% FBS (no phenol red). Following incubation for 24 hours, cells were treated with a final concentration of 0.1% DMSO (vehicle control), 0.1, 1, 10 or 100 μM adamastat in 100 μL of medium and incubated for 96 hours. Subsequently, 40 μL of MTS/PMS solution were added to each well and the absorbance at 490 nm was measured after 2 hours of incubation at 37°C [38]. The assay was performed in triplicate wells. Blank wells, medium only and 2.5 μM staurosporin (Sigma-Aldrich Co.) were included as controls. Cell viability (%) was assess as: (OD sample–OD staurosporine)/(OD medium–OD staurosporine) × 100.

### Tumor Growth in Severe Combined Immunodeficiency (SCID) Mice

To study the effect of silencing ADAM15 on bladder cancer growth, ctlA15 and shA15 UM-UC-6 cells were subcutaneously inoculated at a concentration of 1x10^6^ cells into C57BL/6 SCID mice, in two groups of 10 mice. To determine sample size for the adamastat *in vivo* experiments we used the formula: (n = log 0.10 probability of type II error divided by 95% chance of detecting a weight difference of 40% or more. Log 0.10 divided by log 0.6 = 4.5 mice per group), thus the 5 mice per group used in the experiment. After 3 weeks, the mice were euthanized and tumors weighed. *In vivo* efficacy of adamastat was also determined in UM-UC-6 xenografts, where 1x10^6^ UM-UC-6 bladder tumor cells were injected subcutaneously into the flank of SCID mice. In light of the predicted adamastat oral availability, animals were treated by gavage with adamastat after tumor initiation (10 days after injection). The mice were separated into two groups of 5 animals and treated daily with adamastat (100 mg/kg/dose) or vehicle control (PBS) for 3 weeks before necropsy. Each tumor was resected and weighed at the same time postmortem. The experimental unit was one tumor per mouse and all the tumors (100%) were included in the analysis.

The number of animals included was previously derived by observation of the minimum amount that could present statistical significance under the experimental conditions. All groups were randomly segregated by cage. Cancer cell inoculation or adamastat treatment was started at the same time. The mice did not show weight loss and no adverse effects due to adamastast or the gavage technique were observed.

The SCID mice used in this study were males and females, 8 to 10 weeks old, bred by the ULAM Breeding Core. Animals were housed in a pathogen free facility in ventilated cages. Their welfare was assessed daily by the authors and the veterinary staff of the University of Michigan.

#### Ethics Statement

The care and treatment of the mice was reviewed by the University Committee on Use and Care of Animals (UCUCA) and was found to be in agreement with the University of Michigan institutional guidelines. University of Michigan UCUCA approved these animal studies by protocol PRO00004867. Inoculation of tumor cells was performed under isofluorane anesthesia to minimize suffering. All procedures adhere to the ARRIVE Guidelines for reporting animal research as described in S1 ARRIVE Checklist.

### Statistical Analysis

In tissue microarray analysis, a staining index (intensity score multiplied by the percentage of tumor cells staining) was calculated for each core. Multiple cores of the same clinical stage from the same patient were combined into a summary score representing the mean of the cores. This summary resulted in 48 data points (corresponding to unique patient-diagnosis combinations) for ADAM15 staining evaluation. A one-way ANOVA was used to compare the staining index summary score stage. All pairwise comparisons were made between groups using the Tukey-Kramer method to correct for multiple comparisons. The weakness of the ANOVA model is that there may be correlation within the patient that is not accounted for with this method. A repeated measures model using staining index for each core individually with correlation structures to account for the repeated cores within patient and diagnosis was used to check the results of the ANOVA summary score model. This model broke normality assumptions but had similar results and the same conclusions. For simplicity, ANOVA results are displayed. Unpaired, 2-tailed t-tests were employed to determine if statistically significant differences were observed in wound healing, invasion, migration, and *in vivo* mouse xenograft experiments. Proliferation and cell viability results were evaluated by 2-way ANOVA and Turkey’s multiple comparison tests. Analysis was performed utilizing GraphPad Prism 6 Software (La Jolla, CA). Data were considered statistically significant at *p*<0.05.

## Results

### ADAM15 Expression is Associated with Local Invasion and Metastatic Progression of Human Bladder Cancer

**Fig 1:**
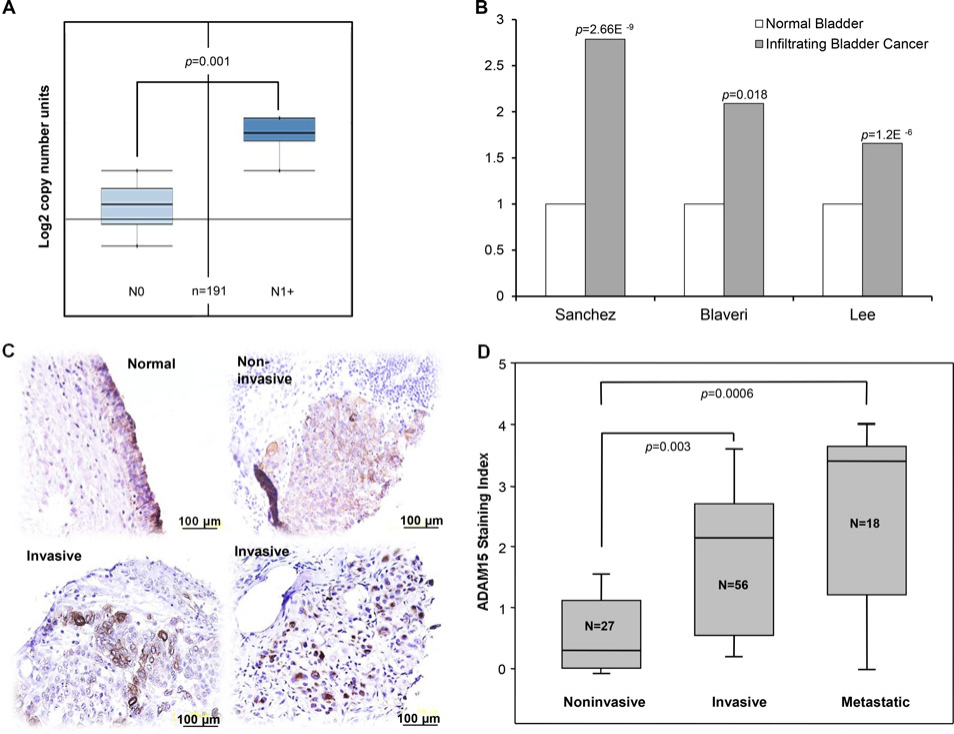
The metastatic progression of human bladder cancer is associated with elevated DNA copy number and increased ADAM15 expression. Analysis utilizing the Oncomine^TM^ (Compendia Bioscience) gene browser. *A)* Boxplots summarize mean copy number and standard deviation (SD) of a bladder cancer gene expression data set. ADAM15 copy number was elevated in invasive bladder cancer (N1+) compared to noninvasive disease (N0). *B)* ADAM15 mRNA is overexpressed in invasive bladder cancer compared to noninvasive bladder cancer. Bars represent ADAM15 mRNA levels in three different published mRNA expression studies. *C)* Microphotographs of ADAM15 immuno-staining of a bladder cancer progression tissue arrays. Three pathological stages are represented (normal tissue, noninvasive, and invasive bladder cancer). *D)* Boxplots represent the ADAM15 staining index in this TMA as mean ± SD. Invasive and metastatic bladder cancer specimens exhibited significantly increase staining index compared to noninvasive disease.

We had previously reported that the expression of ADAM15 was associated with the metastatic progression of breast and prostate cancers [20]. However, the role of ADAM15 in the progression of bladder cancer had not been examined. We began this study by surveying the Oncomine^TM^ (Compendia Bioscience, Ann Arbor, MI) gene expression database to assess DNA copy number and mRNA levels of ADAM15 in human bladder cancer arrays. An analysis of 191 samples revealed that ADAM15 copy number is significantly elevated in advanced N stage (N1+) invasive bladder cancer compared to noninvasive (N0) disease. Of the 18,823 analyzed genes, ADAM15 ranked as the 45^th^ highest in copy number or in the top 5% (Fig 1A). By further analyzing 3 independent transcriptome studies in Oncomine^TM^ (Sanchez, Lee and Blaveri), we found significant overexpression of ADAM15 mRNA in infiltrating bladder cancer compared to normal tissues (Fig 1B).

The significance of these observations to bladder cancer progression was validated by evaluation of ADAM15 protein expression in clinical specimens. Immunostaining of a bladder cancer tissue microarray was performed, showing specific focal overexpression of ADAM15 in the majority of the advanced (invasive and metastatic) bladder cancer specimens. By comparison, all of the low grade and noninvasive bladder cancer samples (27/27), exhibited low ADAM15 staining index (0–2), while 48% (27/56) of the invasive and 72% (13/18) of the metastatic cases exhibited moderate to high staining index (2–4) (Table 1). A closer histological evaluation revealed that ADAM15 localizes in the normal urothelium with a highly organized staining at cell junctions, and increased positivity at the umbrella cell luminal surface (Fig 1C). In contrast, invasive cancer specimens exhibited a more disorganized and cytoplasmic staining of ADAM15. The ADAM15 immuno-positivity increased in advanced stage (Fig 1D) and was significantly greater as the tumors progressed from noninvasive to invasive and metastatic disease. Taken together, these results demonstrated that ADAM15 overexpression is closely associated with the local invasion and metastatic progression of human bladder cancer.

**Table 1:** ADAM15 staining index in bladder cancer microarrays.

	Staining Index				
Pathology	Sample #	0–1	1–2	2–3	3–4
**Noninvasive**	**27**	**21**	**7**	**0**	**0**
**Invasive**	**56**	**18**	**8**	**19**	**8**
**Metastatic**	**18**	**4**	**1**	**3**	**10**

The table represents the ADAM15 immunostaining index in a bladder cancer tissue microarray including noninvasive, invasive and metastatic specimens. Immunostaining index = % of positive cells x staining score.

### Knockdown of ADAM15 Decreases Migration of Bladder Cancer Cells

**Fig 2:**
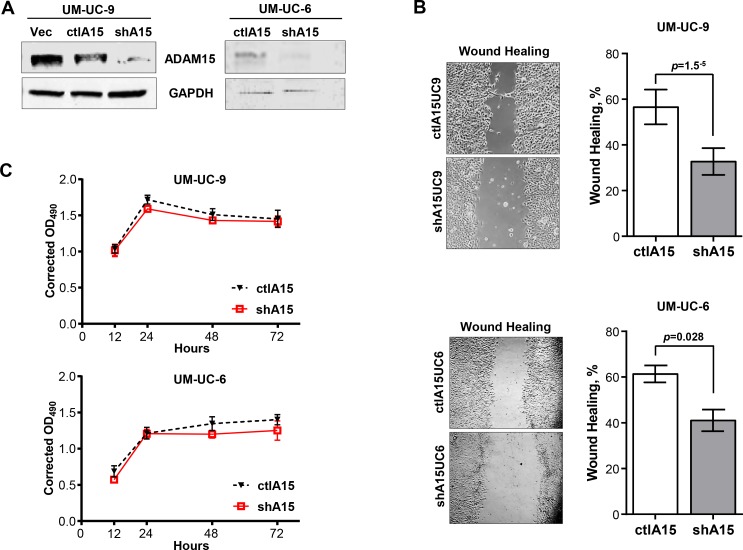
Knockdown of ADAM15 inhibits bladder tumor cell motility. *A)* ADAM15 immunoblot of UM-UC-9 bladder cancer cells expressing empty vector and scrambled ADAM15 sequences (ctl) and short hairpin RNA (shA15) and UM-UC-6 expressing scrambled ADAM15 sequences (ctl) and short hairpin RNA (shA15). Glyceraldehyde-3-phosphate dehydrogenase (GAPDH) was used as loading control. *B)* Wound healing assay comparing extent of wound closure in shA15 UM-UC-9 and shA15 UM-UC-6 cells compared to controls (ctl). Bars represent mean ± SD (n = 4) of one of two independent experiments (UM-UC-9, *top*) and mean ± SEM (N = 3) (UM-UC-6, *bottom*). *C)* Proliferation assays of both knockdown (shA15) and control (ctl) UM-UC-9 and UM-UC-6 cells over a 72 hour time course. Results represent mean ± SD of one of three independent experiments.

Cellular motility is required for tumor invasion and metastasis. We investigated the endogenous expression of ADAM15 in two bladder cancer cell models and analyzed whether the reduction in ADAM15 expression inhibits bladder tumor cell motility. Utilizing the transitional cell carcinoma lines UM-UC-9 and UM-UC-6 [31,32], we assessed ADAM15 protein level by immunoblot analysis using a cytoplasmic-specific antibody against ADAM15. UM-UC-9 and UM-UC-6 bladder cancer cells expressed moderate levels of ADAM15 protein when normalized to GAPDH loading controls (Fig 2A, S1 Fig and S2 Fig). Using a lentiviral RNA inhibitory construct, we decreased the expression of ADAM15 protein in both cell lines (shA15) (Fig 2A, S1 Fig and S2 Fig). Control empty vector and scrambled oligo cells (ctlA15) retained normal expression of ADAM15. Using these cell lines, we performed wound-healing migration assays. Microscopic examination over 24 and 12 hours revealed a 42% reduction (ctlA15UC9 56.58 ± 7.63%, shA15UC9 32.73 ± 5.87%) and a 33% reduction (ctlA15UC6 61.36 ± 3.73%, shA15UC6 41.04 ± 4.72%) in the wound closure of UM-UC-9 and UM-UC-6 ADAM15 knockdown cells respectively compared to the controls (Fig 2B). To rule out anti-proliferative effects of our knockdown and control constructs in the migration assay, we measured cellular proliferation using the MTS chromogenic assay, which revealed no changes in proliferative rates of the shA15 UM-UC-6 or shA15 UM-UC-9 cell lines compared to control lines at 12 and 24 hours (Fig 2C).

### Knockdown of ADAM15 Decreases Bladder Cancer Cell Invasion and Transmigration Through Vascular Endothelium

**Fig 3:**
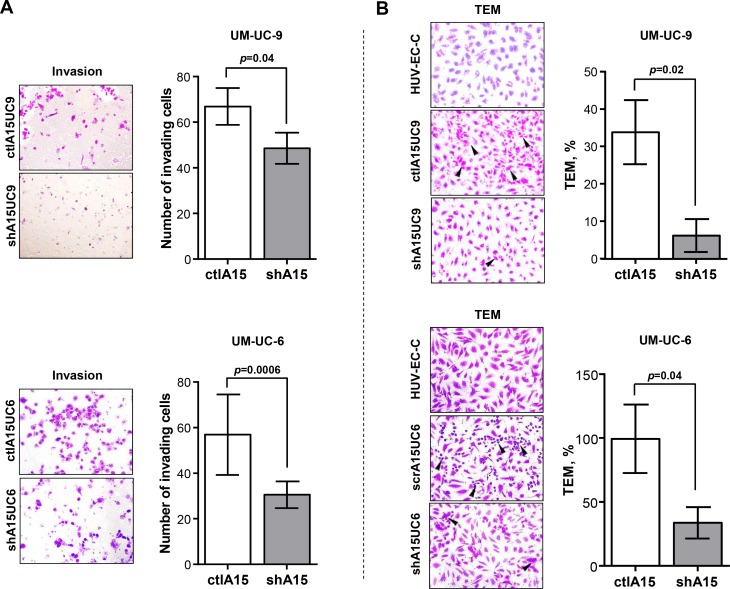
Knockdown of ADAM15 decreases bladder cancer cell invasion and transmigration through vascular endothelium. *A)* Extent of Matrigel^TM^ invasion of UM-UC-9 and UM-UC-6 bladder cancer cells expressing empty vector and scrambled ADAM15 sequences (ctl) and short hairpin RNA (shA15). The bars indicate mean invading cells ± SD (n = 3). The data are representative of 1 of 5 UM-UC-9 or 1 of 3 UM-UC-6 independent experiments. A representative 20X image of each condition is shown. *B)* Transendothelial migration assay revealed reduced transmigration through an endothelial HUV-EC-C monolayer of shA15UM-UC-9 and shA15UM-UC-6 cells when compared with control cells (ctl). Bars represent mean ± SEM (N = 3). A representative 20X image of each condition is shown.

Tumor metastasis to distant tissues is facilitated by the ability of tumor cells to intra and extravasate through vascular endothelium and access the circulation [39,40]. To begin to examine the role of ADAM15 in this process, we evaluated the ADAM15 knockdown cell lines in Matrigel^TM^ invasion assays. The shA15 UM-UC-9 and UM-UC-6 lines revealed a significant reduction of 27.4% and 47.4% respectively in invasive capacity through Matrigel^TM^ when compared to control cells (ctlA15UC9 66.90 ± 8.10 invading cells, shA15UC9 48.60 ± 6.82 invading cells; and ctlA15UC6 56.89 ± 11.67 invading cells, shA15UC6 30.56 ± 5.85 invading cells) (Fig 3A).

A more relevant assay for vascular intravasation is the Trans-Endothelial Migration assay (TEM). We next examined the ability of the shA15 UM-UC-9 and UM-UC-6 cells to transmigrate though monolayers of Human Umbilical Vein Endothelial Cells (HUV-EC-C). The shA15 UM-UC-9 cells were significantly inhibited (81.7%) in their ability to transmigrate through the HUV-EC-C monolayers compared to controls (ctlA15UC9 33.82 ± 8.56%, shA15UC9 6.19 ± 4.42%) (Fig 3B, *top*). The shA15 UM-UC-6 cells were also significantly inhibited (66.1%) in their ability to transmigrate through the HUV-EC-C monolayers compared to controls (ctlA15UC6 99.41 ± 26.74%, shA15UC6 33.68 ± 12.24%) (Fig 3B, *bottom*). These results are consistent with previous reports from our laboratory [22,23] and demonstrate that ADAM15 may play a functional role in bladder cancer migration and vascular trans-migration.

### Specific Inhibition of ADAM15 Catalytic Activity Reduces Bladder Cancer Cell Viability

**Fig 4:**
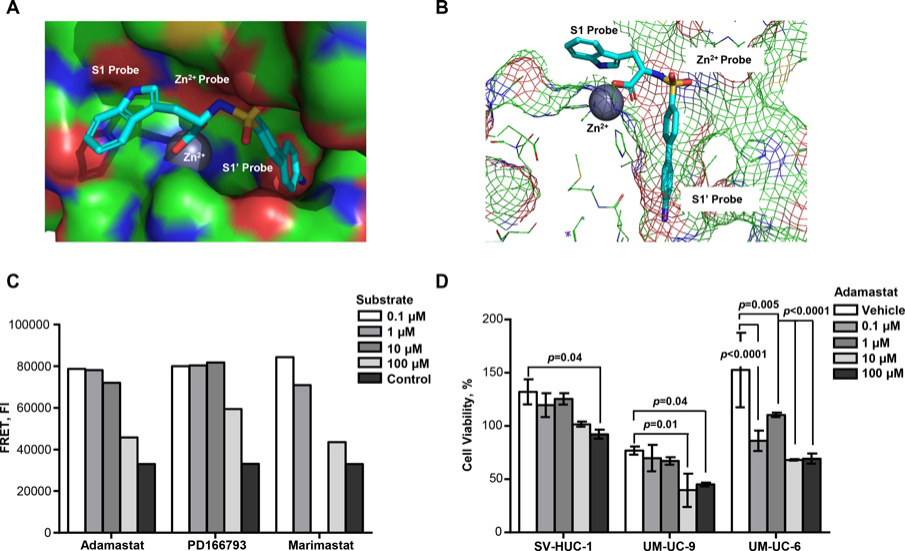
Novel ADAM15-specific sulfonamide inhibitor reduces viability of bladder cancer cells. *A)* Surface model of adamastat binding to the ADAM15 catalytic. *B)* A mesh representation of adamastat binding in the S1’ pocket of the catalybtic domain of ADAM15. *C)* Fluorescence Resonance Energy Transfer assay indicating that inhibition of the recombinant ADAM15 catalytic domain (rA15cat) by adamastat is as effective as two other previously tested metalloproteinase inhibitors over the same concentration range. FI, Fluorescence Intensity. *D)* Viability of adamastat treated SV-HUC-1, UM-UC-9 and UM-UC-6 cells. Vehicle represents 0.1% DMSO control. Bars show mean cell viability % ± SD in 1 out of three independent experiments.

Computational methods using AutoDock Vina software indicated that our ADAM15 catalytic domain inhibitor, adamastat (Fig 4A), binds to the active site of the ADAM15 metalloproteinase domain with a calculated binding energy of -9.0 kcal/mol (S1 Table). This preferred binding mode places the biphenyl amino side chain of the inhibitor in the deep amphipathic S1’ amino acid recognition pocket of the ADAM15 catalytic domain (Fig 4B). The inhibitory properties of adamastat were verified using a Fluorescence Resonance Energy Transfer (FRET) assay that measures the cleavage of fluorogenic ADAM15 substrate peptides. Inhibition of the recombinant ADAM15 catalytic domain (rA15cat) by adamastat was compared to previously validated and clinically tested metalloproteinase inhibitors, marimastat and PD166793. The result of this analysis indicated that adamastat is as effective over a 100 fold molar range as the clinically tested compounds, PD166793 and marimastat (Fig 4C).

To examine the effect of adamastat on human bladder cancer cells, we treated UM-UC-9 and UM-UC-6 bladder cancer cells and SV-HUC-1 immortalized bladder urothelial cells with increasing concentrations (0.1–100 μM) of adamastat and measured cell viability. Adamastat significantly inhibited the viability of UM-UC-9 and UM-UC-6 cells at 10 μM (Fig 4D). Interestingly, UM-UC-6 cells more sensitive, exhibiting significant reduction in cell viability at 1 μM (Fig 4D). Adamastat demonstrated very little effect was observed on the immortalized non-cancerous SV-HUC-1 cells (Fig 4D). We also examined the effects of adamastat on the proliferation rates of the three cell lines and found that adamastat does not affect proliferation of any cell line compared to the DMSO vehicle (S3 Fig). Collectively, these data suggest that targeted inhibition of ADAM15 catalytic activity by adamastat reduces the viability of human bladder cancer cells. As a control for off-target effects, we calculated binding affinities of all three inhibitors (adamastat, marimastat and PD166793) for the active sites of 4 other critical metalloproteinases related to ADAM15 using the AutoDock Vina software. These calculations revealed that adamastat binding affinity would be most selective for ADAM15 in comparison to other ADAM family members and MMPs. Marimastat and PD166793 showed little preference for any of the metalloproteinases tested (S1 Table).

### The Inhibition of ADAM15 Activity Reduces Tumor Growth *In Vivo*

**Fig 5:**
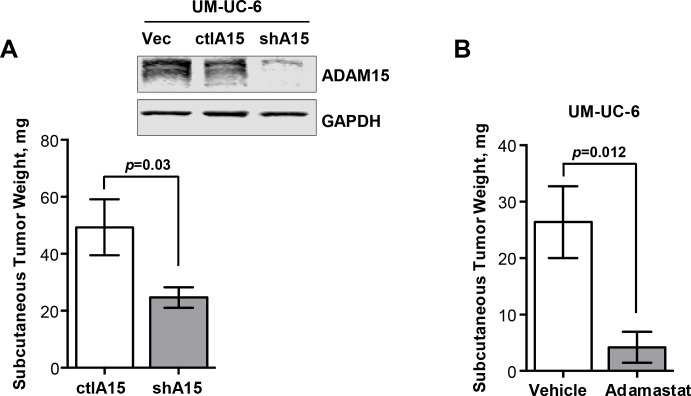
*In vivo* inhibition of ADAM15 activity reduces tumor growth. *A)* ADAM15 immunoblot of UM-UC-6 bladder cancer cells expressing empty vector (Vec), control scrambled ADAM15 sequences (ctlA15UC6) and short hairpin RNA (shRNA) sequence (shA15UC6). GAPDH was utilized as loading control (*top*). Subcutaneous inoculation of shA15UC6 cells (n = 10) in SCID mice led to decreased tumor weight compared to ctlA15UC6 control cells (n = 10) (*bottom*). *B)* UM-UC-6 cells were subcutaneously implanted in SCID mice. Specific inhibition of the ADAM15 catalytic activity by daily treatment with adamastat 100 mg/kg (n = 5) during 3 weeks led to decrease in tumor growth compared to vehicle treated mice (n = 5). Bars represent mean tumor weight (mg) ± SD (*5A*,*B*).

To investigate the effect of ADAM15 ablation in tumor development, we employed our UM-UC-6 model using the same silencing shRNA sequences and procedures described in the methods section. Immunoblot analysis confirmed reduced protein expression of ADAM15 in UM-UC-6 knockdown (shA15) compared to vector only (Vec) and scrambled control cells (ctlA15) (Fig 5A and S4 Fig). We then inoculated ctlA15UC6 and shA15UC6 cells subcutaneously in SCID mice and evaluated the tumor weights after 2 weeks. The knockdown of ADAM15 decreased the UM-UC-6 tumor growth by 44.5% (ctlA15UC6 49.9±9.81mg, shA15UC6 24.7±3.60mg) (Fig 5A). Lastly, we grafted UM-UC-6 cells subcutaneously in SCID mice and treated the mice daily by gavage with vehicle control or 100 mg/kg adamastat for 3 weeks. In this experiment, adamastat reduced tumor growth by 83.8% (vehicle control 26.4±14.2 mg, adamastat 4.2±6.1 mg) (Fig 5B). These results support the *in vivo* relevance of ADAM15 and its metalloproteinase activity to tumor development.

## Discussion

Previous studies have speculated on the role of ADAMs in human tumorigenesis, cancer progression and aggressiveness [13,14,16,18,20,21]. ADAM15 is a multi-domain disintegrin metalloproteinase that is upregulated in several solid malignancies including prostate and breast cancer and elevated expression of ADAM15 has also been correlated with the metastatic progression of these tumors [19–21]. Elevated ADAM15 mRNA expression has also been associated with poor patient outcome as it predicts a shorter relapse-free survival in lymph node-negative breast cancer [41]. Although suggestive in other tumors, the association of ADAM15 with the instance or progression of human bladder cancer has not been previously investigated.

Using meta-analysis of various expression arrays, we previously demonstrated significant overexpression of ADAM15 in independent bladder cancer gene expression studies [21]. The present study examined ADAM15 copy number, mRNA levels and protein expression in clinical specimens of bladder cancer, which revealed significant correlations between amplification of the ADAM15 gene and overexpression of ADAM15 mRNA and protein in the progression of this disease. This study also revealed that overexpression of ADAM15 correlated with tumor invasion and increased bladder cancer stage, suggesting a contributing role of ADAM15 in the progression of human bladder cancer.

The molecular mechanism by which ADAM15 could support bladder cancer progression is unknown. ADAM15 is associated with diverse biological functions in cancer progression and metastasis, such as neovascularization, tissue-remodeling, reduction in cell-cell adhesions and the release of various cytokines and growth factors that may promote tumor growth and survival [19,24,25]. We have previously demonstrated that siRNA targeting of ADAM15 in prostate cancer cells not only reduced tumor cell migration and invasion, it also altered the tumor cell adhesion profile and completely inhibited the overt metastasis of these cells *in vivo* [22,23]. The local invasion and vascular intravasation and extravasation are required for tumor cell dissemination in metastasis [40,42,43]. In examining disease progression tumor arrays of prostate and breast cancer, we found a significant correlation between elevated ADAM15 levels and angio-invasive breast cancer [20]. Previous reports have linked ADAM15 with regulation of endothelial permeability and neutrophil and monocyte transmigration during vascular disease and inflammation [44–46]. Herein we have shown that the knockdown of ADAM15 lead to *in vitro* reduction of the migratory and invasive properties of human bladder cancer cells, including the ability to transmigrate though vascular endothelial monolayers. Taken together, these data suggest a functional role of ADAM15 in the remodeling of the extracellular matrix, local invasion and interaction with the vascular endothelium during the metastatic progression of human bladder cancer.

Our previous work has also demonstrated that ADAM15 can proteolytically process different molecules to modulate cell adhesion and cancer cell metastasis [19]. To test whether the proteolytic function of ADAM15 is relevant in the regulation of bladder cancer microenvironment, we designed and synthesized adamastat, a selective inhibitor of ADAM15 metalloproteinase catalytic activity. Here, we demonstrated that adamastat triggers the reduction of bladder cancer cell viability. Although PD166793 and marimastat have been shown to block tumor growth, the inhibition of their metalloproteinase activity has not been linked with cellular cytotoxicity [47–51].

ADAM15 has been implicated in resistance to apoptosis [52,53] and the regulation of multiple signaling pathways involved in cell survival and proliferation [14,19,54]. Several substrates of ADAM15, including EGFR ligands such as HB-EGF, TGFα, amphiregulin, betacellulin, and other factors including Notch, and cytokines have been identified in cancer cells [10,12,15,16,54,55]. ADAM15 has also been implicated in stimulation of G-protein coupled receptor (GPCR)-dependent shedding of EGFR ligands in multiple systems [56]. We previously reported that an unknown proteolytic mechanism resulted in the shedding of the extracellular domain of E-cadherin into the serum of patients with metastatic prostate cancer [57,58], also known as soluble E-cadherin (sE-cad). We also discovered that ADAM15 expression in metastatic prostate cancer correlated with elevated levels of sE-cad found in the serum of these patients [57,58] and demonstrated that ADAM15 did indeed cleave E-cadherin and N-cadherin in tumor cells [19,21–23].

Cadherins are type I transmembrane glycoproteins that play important roles in cell adhesion [59]. E-cadherin is recognized as a tumor suppressor given its ability to inhibit cellular migration and invasion [60,61]. The disruption of cellular E-cadherin, specifically by proteolytic cleavage, causes loss of cell-cell adhesion. Cleavage of E-cadherin causes release of a smaller 90 kDa fragment from the extracellular domain that both inhibits intact E-cadherin function [62], and promotes EGFR activation [21,22]. Ectodomain shedding of a stable soluble E-cadherin fragment has been shown to be increased in the urine and serum of patients of multiple carcinomas [26,27,63] including bladder cancer, where increased sE-cad levels also correlate with higher stage disease and poorer outcome [26,27]. These findings suggest that activated ADAM15 may be associated with E-cadherin shedding in such patients, increasing cell signaling as well as decreasing cell-cell binding and thus allowing bladder cancer progression. However, further research is needed to assess the interactions of ADAM15 and sEcad in advanced bladder cancer.

The *in vivo* significance of reduced ADAM15 activity was analyzed in xenograft models of transitional cell carcinoma of the bladder. In these experiments, both ADAM15 knockdown or inhibition of its catalytic activity by adamastat proved to reduce bladder tumor growth in immuno-deficient mice; however, a full histopathological assessment as to whether the reduced tumor size is due to cytotoxicity remains to be evaluated. The reduction of tumor growth and metastasis by loss of ADAM15 has also been reported by our laboratory and others using different xenograft tumor models [14,19,23,25]. Collectively these findings lend further support for ADAM15, and in particular its catalytic function, in the metastatic progression of human bladder cancer.

In conclusion, we have found a novel association between ADAM15 expression and invasive bladder cancer and provide functional evidence for the role of ADAM15 in this disease. This is supported by our *in vitro* observations and early *in vivo* results by which the reduction of ADAM15 expression or inhibition of its catalytic activity, reduces cellular motility and invasiveness and inhibits tumor growth in animal models. Taken together, these results now suggest a critical role of ADAM15 and its metalloproteinase activity during the progression of human bladder cancer and potential therapeutic target.

## Supporting Information

S1 ARRIVE Checklist ARRIVE guidelines checklist. (PDF)

S1 Fig ADAM15 protein expression in UM-UC-9 bladder cancer cells. (TIFF)

S2 Fig ADAM15 protein expression in UM-UC-6 bladder cancer cells. (TIFF)

S3 Fig Effects of adamastat on cell proliferation. (TIFF)

S4 Fig ADAM15 protein expression in xenografted UM-UC-6 bladder cancer cells. (TIFF)

S1 Table Calculated affinities of adamastat and metalloproteinase inhibitors. (PDF)

## Abbreviations

ADAM: A Disintegrin And Metalloproteinase
DMEM: Dulbecco’s Modified Eagle Medium
EGF: Epidermal Growth Factor
EGFR: Epidermal Growth Factor Receptor
FBS: Fetal Bovine Serum
FRET: Fluorescence Resonance Transfer
GAPDH: Glyceraldehyde-3-Phosphate Dehydrogenase
GPCR: G-protein Coupled Receptor
HB-EGF: Heparin Binding Epidermal Growth Factor
HUV-EC-C: Human Umbilical Vein Endothelial Cells
MTS: 3-(4,5-dimethylthiazol-2-yl)-5-(3-carboxymethoxyphenyl)-2-(4-sulfophenyl)-2H-tetrazolium
OD: Optical Density
PBS: Phosphate Buffered Saline
PMS: Phenazine Methosulfate
RFLP: fragment length polymorphism
SCID: Severe Combined Immunodeficiency
SD: Standard Deviation
sE-cad: soluble E-cadherin
SEM: Standard Error of the Mean
shRNA: short hairpin RNA
TBST: Tris-Buffered Saline plus Tween
TEM: Trans-Endothelial Migration
TGFα: Transforming Growth Factor Alpha
TGFβ: Transforming Growth Factor Beta

## References

[1] Siegel RL, Miller KD, Jemal A. Cancer statistics, 2015. CA Cancer J Clin. 2015;65(1): 5–29. 10.3322/caac.21254 25559415

[2] van Rhijn BW, Burger M, Lotan Y, Solsona E, Stief CG, Sylvester RJ, et al Recurrence and progression of disease in non-muscle-invasive bladder cancer: from epidemiology to treatment strategy. Eur Urol. 2009;56(3): 430–42. 10.1016/j.eururo.2009.06.028 19576682

[3] Dreicer R. Advanced bladder cancer: so many drugs, so little progress: what's wrong with this picture? Cancer. 2008;113(6): 1275–7. 10.1002/cncr.23690 18615663

[4] Hussain MH, Wood DP, Bajorin DF, Bochner BH, Dreicer R, Lamm DL, et al Bladder cancer: narrowing the gap between evidence and practice. J Clin Oncol. 2009;27(34): 5680–4. 10.1200/JCO.2009.23.6901 19858384 PMC4659855

[5] Mellon K, Wright C, Kelly P, Horne CH, Neal DE. Long-term outcome related to epidermal growth factor receptor status in bladder cancer. J Urol. 1995;153: 919–25. 7853575

[6] Neal DE, Marsh C, Bennett MK, Abel PD, Hall RR, Sainsbury JR, et al Epidermal-growth-factor receptors in human bladder cancer: comparison of invasive and superficial tumours. Lancet. 1985;1(8425): 366–8. 2857420 10.1016/s0140-6736(85)91386-8

[7] Kramer C, Klasmeyer K, Bojar H, Schulz WA, Ackermann R, Grimm MO. Heparin-binding epidermal growth factor-like growth factor isoforms and epidermal growth factor receptor/ErbB1 expression in bladder cancer and their relation to clinical outcome. Cancer. 2007; 109: 2016–24. 17394193 10.1002/cncr.22627

[8] Colquhoun AJ, Mellon JK. Epidermal growth factor receptor and bladder cancer. Postgrad Med J. 2002;78(924): 584–9. 12415079 10.1136/pmj.78.924.584 PMC1742539

[9] Black PC, Dinney CP. Bladder cancer angiogenesis and metastasis—translation from murine model to clinical trial. Cancer Metastasis Rev. 2007;26(3–4): 623–34. 17726580 10.1007/s10555-007-9084-9

[10] Schäfer B, Gschwind A, Ullrich A. Multiple G-protein-coupled receptor signals converge on the epidermal growth factor receptor to promote migration and invasion. Oncogene. 2004;23(4): 991–9. 14647423 10.1038/sj.onc.1207278

[11] Bue P, Wester K, Sjöström A, Holmberg A, Nilsson S, Carlsson J, et al Expression of epidermal growth factor receptor in urinary bladder cancer metastases. Int J Cancer. 1998;76: 189–93. 9537579 10.1002/(sici)1097-0215(19980413)76:2<189::aid-ijc4>3.0.co;2-t

[12] Blobel CP. ADAMs: key components in EGFR signalling and development. Nat Rev Mol Cell Biol. 2005;6(1): 32–43. 15688065 10.1038/nrm1548

[13] Duffy MJ, McKiernan E, O'Donovan N, McGowan PM. Role of ADAMs in cancer formation and progression. Clin Cancer Res. 2009;15(4): 1140–4. 10.1158/1078-0432.CCR-08-1585 19228719

[14] Duffy MJ, Mullooly M, O'Donovan N, Sukor S, Crown J, Pierce A, et al The ADAMs family of proteases: new biomarkers and therapeutic targets for cancer? Clin Proteomics. 2011;8(1): 9 10.1186/1559-0275-8-9 21906355 PMC3170276

[15] Reiss K, Ludwig A, Saftig P. Breaking up the tie: disintegrin-like metalloproteinases as regulators of cell migration in inflammation and invasion. Pharmacol Ther. 2006;111(3): 985–1006. 16626807 10.1016/j.pharmthera.2006.02.009

[16] Huovila AP, Turner AJ, Pelto-Huikko M, Kärkkäinen I, Ortiz RM. Shedding light on ADAM metalloproteinases. Trends Biochem Sci. 2005;30(7): 413–22. 15949939 10.1016/j.tibs.2005.05.006

[17] Edwards DR, Handsley MM, Pennington CJ. The ADAM metalloproteinases. Mol Aspects Med. 2008 10;29(5):258–89. Epub 2008 Aug 15. 10.1016/j.mam.2008.08.001 18762209 PMC7112278

[18] Mochizuki S, Okada Y. ADAMs in cancer cell proliferation and progression. Cancer Sci. 2007 5;98(5):621–8. 17355265 10.1111/j.1349-7006.2007.00434.x PMC11160018

[19] Lucas N, Day ML. The role of the disintegrin metalloproteinase ADAM15 in prostate cancer progression. J Cell Biochem. 2009 4 15;106(6):967–74. 10.1002/jcb.22087 19229865

[20] Kuefer R, Day KC, Kleer CG, Sabel MS, Hofer MD, Varambally S, et al ADAM15 disintegrin is associated with aggressive prostate and breast cancer disease. Neoplasia. 2006;8(4): 319–29. 16756724 10.1593/neo.05682 PMC1600681

[21] Lucas N, Najy AJ, Day ML. The therapeutic potential of ADAM15. Curr Pharm Des. 2009;15: 2311–8. 19601833 10.2174/138161209788682370

[22] Najy AJ, Day KC, Day ML. The ectodomain shedding of E-cadherin by ADAM15 supports ErbB receptor activation. J Biol Chem. 2008;283(26): 18393–401. 10.1074/jbc.M801329200 18434311 PMC2440598

[23] Najy AJ, Day KC, Day ML. ADAM15 supports prostate cancer metastasis by modulating tumor cell-endothelial cell interaction. Cancer Res. 2008;68(4): 1092–9. 10.1158/0008-5472.CAN-07-2432 18281484

[24] Xie B, Shen J, Dong A, Swaim M, Hackett SF, Wyder L, et al An Adam15 amplification loop promotes vascular endothelial growth factor-induced ocular neovascularization. FASEB J. 2008;22: 2775–83. 10.1096/fj.07-099283 18381816 PMC2493454

[25] Horiuchi K, Weskamp G, Lum L, Hammes HP, Cai H, Brodie TA, et al Potential role for ADAM15 in pathological neovascularization in mice. Mol Cell Biol. 2003;23: 5614–24. 12897135 10.1128/MCB.23.16.5614-5624.2003 PMC166329

[26] Shariat SF, Matsumoto K, Casella R, Jian W, Lerner SP. Urinary levels of soluble e-cadherin in the detection of transitional cell carcinoma of the urinary bladder. Eur Urol. 2005;48: 69–76. 15967254 10.1016/j.eururo.2005.02.012

[27] Matsumoto K, Shariat SF, Casella R, Wheeler TM, Slawin KM, Lerner SP. Preoperative plasma soluble E-cadherin predicts metastases to lymph nodes and prognosis in patients undergoing radical cystectomy. J Urol. 2003;170: 2248–52. 14634390 10.1097/01.ju.0000094189.93805.17

[28] Shariat SF, Youssef RF, Gupta A, Chade DC, Karakiewicz PI, Isbarn H, et al Association of angiogenesis related markers with bladder cancer outcomes and other molecular markers. J Urol. 2010;183: 1744–50. 10.1016/j.juro.2010.01.018 20299037

[29] Crew JP, O'Brien T, Bradburn M, Fuggle S, Bicknell R, Cranston D, et al Vascular endothelial growth factor is a predictor of relapse and stage progression in superficial bladder cancer. Cancer Res. 1997;57(23): 5281–5. 9393750

[30] Wu W, Shu X, Hovsepyan H, Mosteller RD, Broek D. VEGF receptor expression and signaling in human bladder tumors. Oncogene. 2003; 22: 3361–70. 12776187 10.1038/sj.onc.1206285

[31] Grossman HB, Wedemeyer G, Ren L, Wilson GN, Cox B. Improved growth of human urothelial carcinoma cell cultures. J Urol. 1986;136(4): 953–9. 3761468 10.1016/s0022-5347(17)45139-1

[32] Shinohara N, Liebert M, Wedemeyer G, Chang JH, Grossman HB. Evaluation of multiple drug resistance in human bladder cancer cell lines. J Urol. 1993;150(2 Pt 1): 505–9. 8100862 10.1016/s0022-5347(17)35536-2

[33] Meisner LF, Wu SQ, Christian BJ, Reznikoff CA. Cytogenetic instability with balanced chromosome changes in an SV40 transformed human uroepithelial cell line. Cancer Res. 1988;48(11): 3215–20. 2835156

[34] Schneider CA, Rasband WS, Eliceiri KW. NIH Image to ImageJ: 25 years of image analysis. Nat Methods. 2012;9(7): 671–5. 22930834 10.1038/nmeth.2089 PMC5554542

[35] O'Brien PM, Ortwine DF, Pavlovsky AG, Picard JA, Sliskovic DR, Roth BD, et al Structure-activity relationships and pharmacokinetic analysis for a series of potent, systemically available biphenylsulfonamide matrix metalloproteinase inhibitors. J Med Chem. 2000;43(2): 156–66. 10649971 10.1021/jm9903141

[36] Trott O, Olson AJ. AutoDock Vina: improving the speed and accuracy of docking with a new scoring function, efficient optimization, and multithreading. J Comput Chem. 2010;31(2): 455–61. 10.1002/jcc.21334 19499576 PMC3041641

[37] Moss ML, Rasmussen FH. Fluorescent substrates for the proteinases ADAM17, ADAM10, ADAM8, and ADAM12 useful for high-throughput inhibitor screening. Anal Biochem. 2007;366(2): 144–8. 17548045 10.1016/j.ab.2007.04.043

[38] Berridge MV, Herst PM, Tan AS. Tetrazolium dyes as tools in cell biology: new insights into their cellular reduction. Biotechnol Annu Rev. 2005;11: 127–52. 16216776 10.1016/S1387-2656(05)11004-7

[39] Chambers AF, Groom AC, MacDonald IC. Dissemination and growth of cancer cells in metastatic sites. Nat Rev Cancer. 2002;2(8): 563–572. 12154349 10.1038/nrc865

[40] Reymond N, d'Água BB, Ridley AJ. Crossing the endothelial barrier during metastasis. Nat Rev Cancer. 2013;13(12): 858–70. 10.1038/nrc3628 24263189

[41] Zhong JL, Poghosyan Z, Pennington CJ, Scott X, Handsley MM, Warn A, et al Distinct Functions of Natural ADAM-15 Cytoplasmic Domain Variants in Human Mammary Carcinoma. Mol Cancer Res. 2008,6: 383–394. 10.1158/1541-7786.MCR-07-2028 18296648

[42] García-Román J, Zentella-Dehesa A. Vascular permeability changes involved in tumor metastasis. Cancer Lett. 2013;335(2): 259–69. 10.1016/j.canlet.2013.03.005 23499893

[43] Labelle M, Hynes RO. The initial hours of metastasis: the importance of cooperative host-tumor cell interactions during hematogenous dissemination. Cancer Discov. 2012;2(12): 1091–9. 10.1158/2159-8290.CD-12-0329 23166151 PMC3540992

[44] Sun C, Wu MH, Guo M, Day ML, Lee ES, Yuan SY. ADAM15 regulates endothelial permeability and neutrophil migration via Src/ERK1/2 signalling. Cardiovasc Res. 2010;87(2): 348–55. 10.1093/cvr/cvq060 20189953 PMC2895539

[45] Sun C, Wu MH, Lee ES, Yuan SY. A disintegrin and metalloproteinase 15 contributes to atherosclerosis by mediating endothelial barrier dysfunction via Src family kinase activity. Arterioscler Thromb Vasc Biol. 2012;32(10): 2444–51. 10.1161/ATVBAHA.112.252205 22904271 PMC3482138

[46] Chatterjee V, Beard RS Jr, Reynolds JJ, Haines R, Guo M, Rubin M, et al MicroRNA-147b regulates vascular endothelial barrier function by targeting ADAM15 expression. PLoS One. 2014;9(10): e110286 10.1371/journal.pone.0110286 25333931 PMC4198252

[47] Watson SA, Morris TM, Collins HM, Bawden LJ, Hawkins K, Bone EA. Inhibition of tumour growth by marimastat in a human xenograft model of gastric cancer: relationship with levels of circulating CEA. Br J Cancer. 1999;81(1): 19–23. 10487607 10.1038/sj.bjc.6690645 PMC2374341

[48] Rasmussen HS, McCann PP. Matrix metalloproteinase inhibition as a novel anticancer strategy: a review with special focus on batimastat and marimastat. Pharmacol Ther. 1997;75(1): 69–75. 9364582 10.1016/s0163-7258(97)00023-5

[49] Hidalgo M, Eckhardt SG. Development of matrix metalloproteinase inhibitors in cancer therapy. J Natl Cancer Inst. 2001;93(3): 178–93. 11158186 10.1093/jnci/93.3.178

[50] Belotti D, Paganoni P, Giavazzi R. MMP inhibitors: experimental and clinical studies. Int J Biol Markers. 1999;14(4): 232–8. 10669951 10.1177/172460089901400406

[51] Mendes O, Kim HT, Stoica G. Expression of MMP2, MMP9 and MMP3 in breast cancer brain metastasis in a rat model. Clin Exp Metastasis. 2005;22(3): 237–46. 16158251 10.1007/s10585-005-8115-6

[52] Böhm BB, Freund I, Krause K, Kinne RW, Burkhardt H. ADAM15 adds to apoptosis resistance of synovial fibroblasts by modulating focal adhesion kinase signaling. Arthritis Rheum. 2013;65(11): 2826–34. 10.1002/art.38109 23918525

[53] Fried D, Böhm BB, Krause K, Burkhardt H. ADAM15 protein amplifies focal adhesion kinase phosphorylation under genotoxic stress conditions. J Biol Chem. 2012;287(25): 21214–23. 10.1074/jbc.M112.347120 22544741 PMC3375543

[54] Schäfer B, Marg B, Gschwind A, Ullrich A. Distinct ADAM metalloproteinases regulate G protein-coupled receptor-induced cell proliferation and survival. J Biol Chem. 2004;279(46): 47929–38. 15337756 10.1074/jbc.M400129200

[55] Hart S, Fischer OM, Prenzel N, Zwick-Wallasch E, Schneider M, Hennighausen L, et al GPCR-induced migration of breast carcinoma cells depends on both EGFR signal transactivation and EGFR-independent pathways. Biol Chem. 2005;386(9): 845–55. 16164409 10.1515/BC.2005.099

[56] Ohtsu H, Dempsey PJ, Eguchi S. ADAMs as mediators of EGF receptor transactivation by G protein-coupled receptors. Am J Physiol Cell Physiol. 2006;291(1): C1–10. 16769815 10.1152/ajpcell.00620.2005

[57] Kuefer R, Hofer MD, Gschwend JE, Pienta KJ, Sanda MG, Chinnaiyan AM, et al The role of an 80 kDa fragment of E-cadherin in the metastatic progression of prostate cancer. Clin Cancer Res. 2003;9(17): 6447–52. 14695147

[58] Kuefer R, Hofer MD, Zorn CS, Engel O, Volkmer BG, Juarez-Brito MA, et al Assessment of a fragment of e-cadherin as a serum biomarker with predictive value for prostate cancer. Br J Cancer. 2005;92(11): 2018–23. 15870707 10.1038/sj.bjc.6602599 PMC2361796

[59] van Roy F, Berx G. The cell-cell adhesion molecule E-cadherin. Cell Mol Life Sci. 2008;65(23): 3756–88. 10.1007/s00018-008-8281-1 18726070 PMC11131785

[60] Masterson J, O'Dea S. Posttranslational truncation of E-cadherin and significance for tumour progression. Cells Tissues Organs. 2007;185(1–3): 175–9. 17587823 10.1159/000101318

[61] Jeanes A, Gottardi CJ, Yap AS. Cadherins and cancer: how does cadherin dysfunction promote tumor progression? Oncogene. 2008;27(55): 6920–9. 10.1038/onc.2008.343 19029934 PMC2745643

[62] Wheelock MJ, Buck CA, Bechtol KB, Damsky CH. Soluble 80-kd fragment of cell-CAM 120/80 disrupts cell-cell adhesion. J Cell Biochem. 1987;34(3): 187–202. 3611200 10.1002/jcb.240340305

[63] De Wever O, Derycke L, Hendrix A, De Meerleer G, Godeau F, Depypere H, et al Soluble cadherins as cancer biomarkers. Clin Exp Metastasis. 2007;24(8): 685–97. 17952616 10.1007/s10585-007-9104-8

