# Supplementary figures and images for: ADAM15 Is Functionally Associated with the Metastatic Progression of Human Bladder Cancer
Source: PLoS One. 2016 Mar 1;11(3):e0150138. doi: 10.1371/journal.pone.0150138 (PMC4773041; doi:10.1371/journal.pone.0150138)

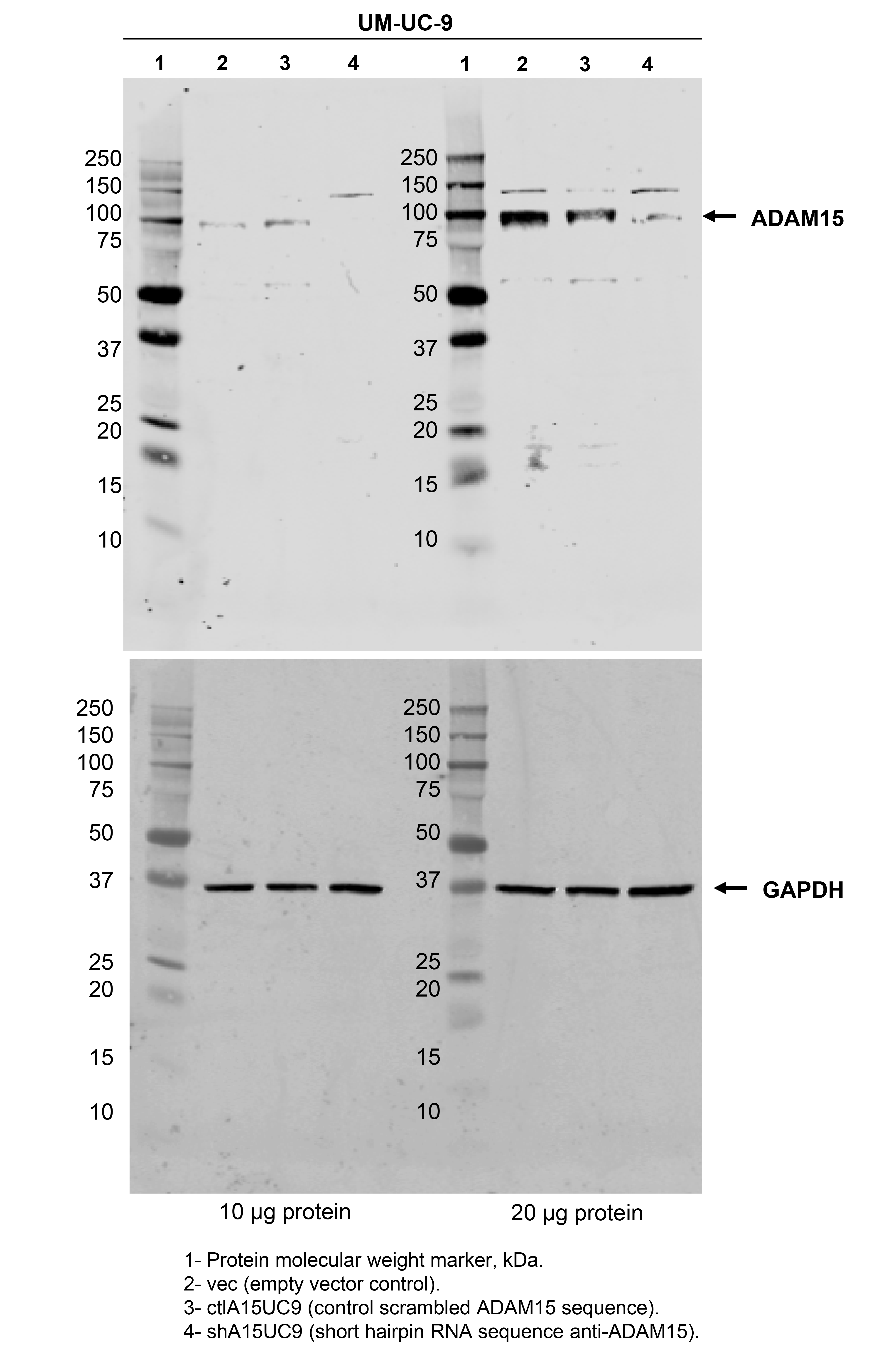

Supplement: S1 Fig — (TIFF) [file pone.0150138.s002.tiff]

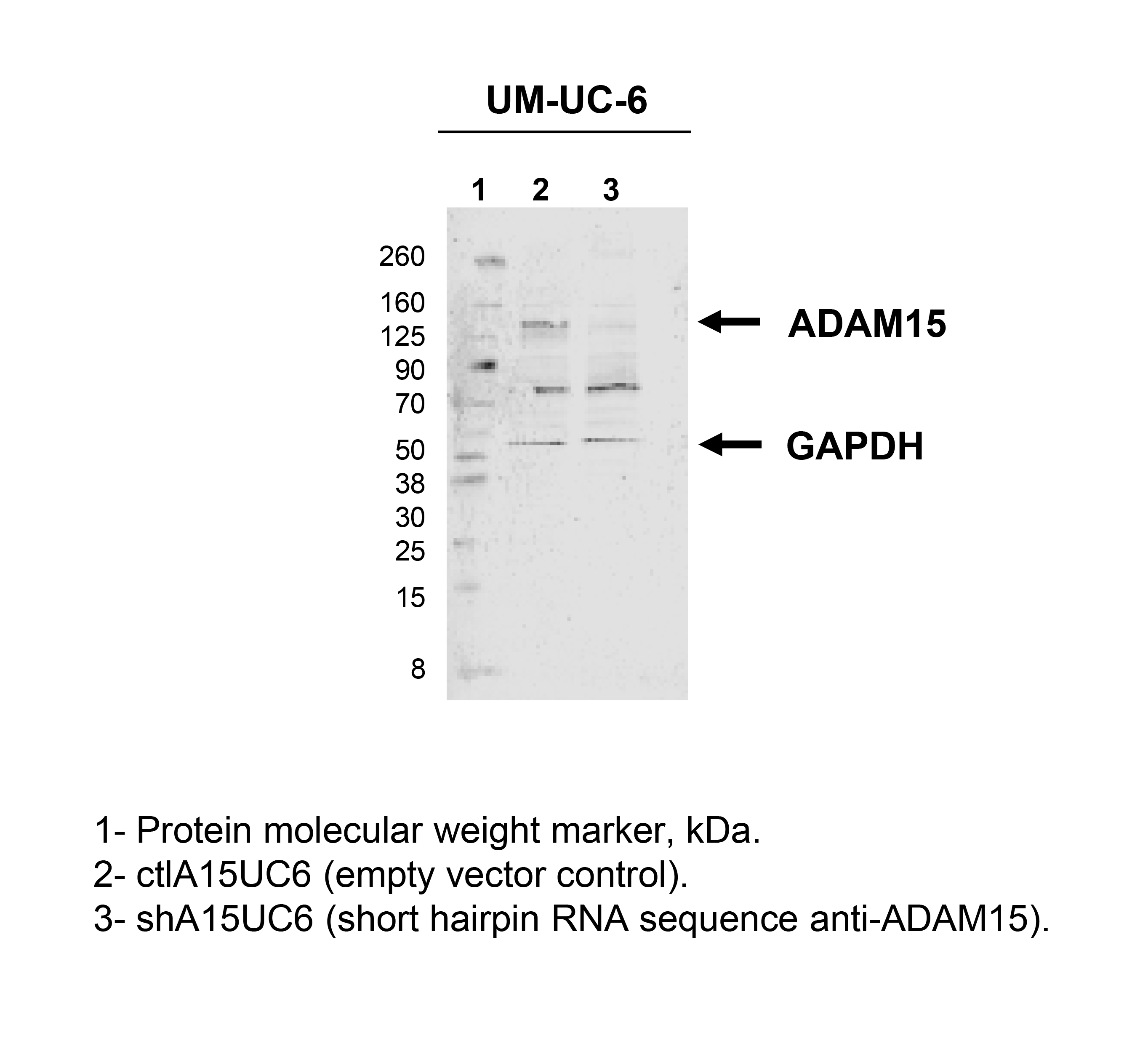

Supplement: S2 Fig — (TIFF) [file pone.0150138.s003.tiff]

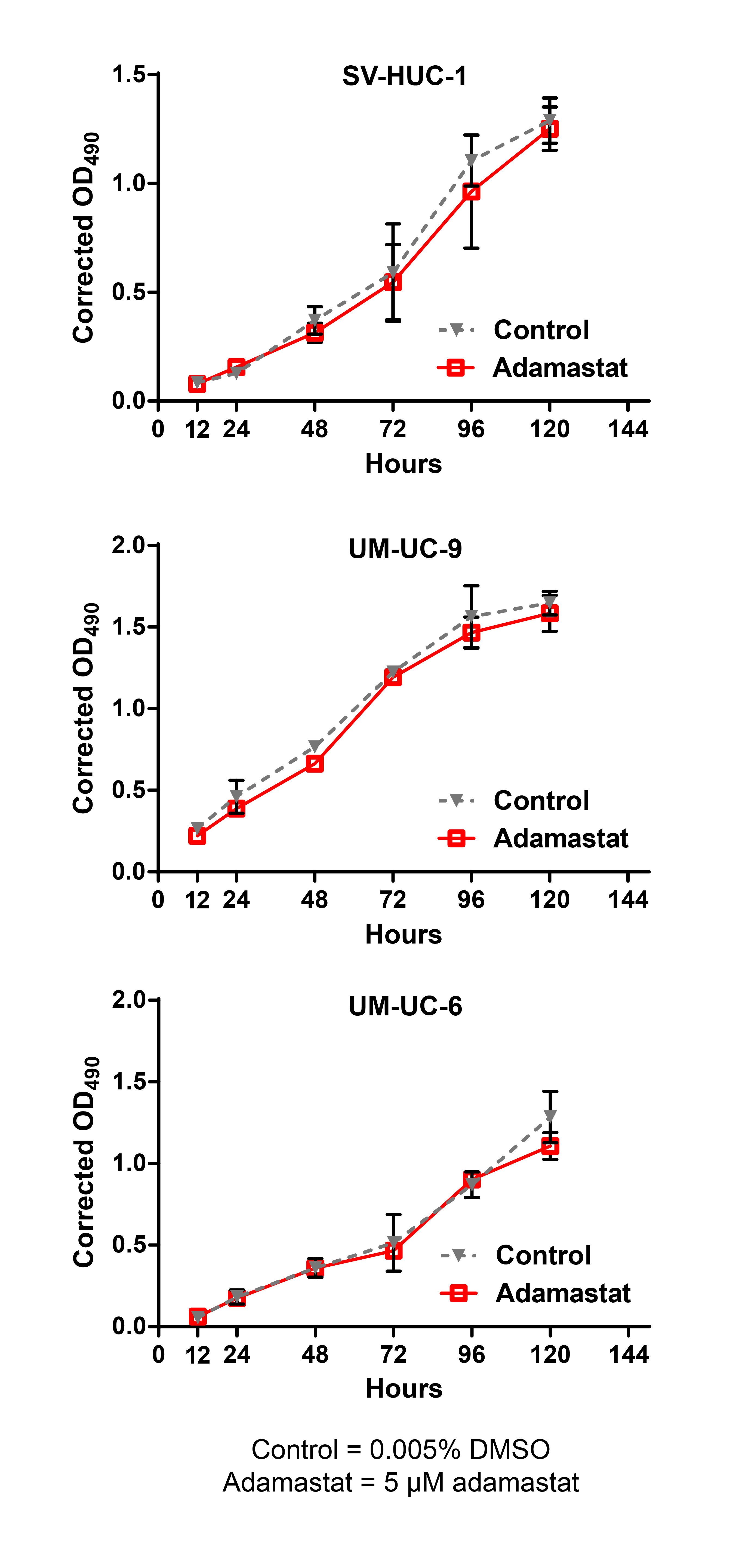

Supplement: S3 Fig — (TIFF) [file pone.0150138.s004.tiff]

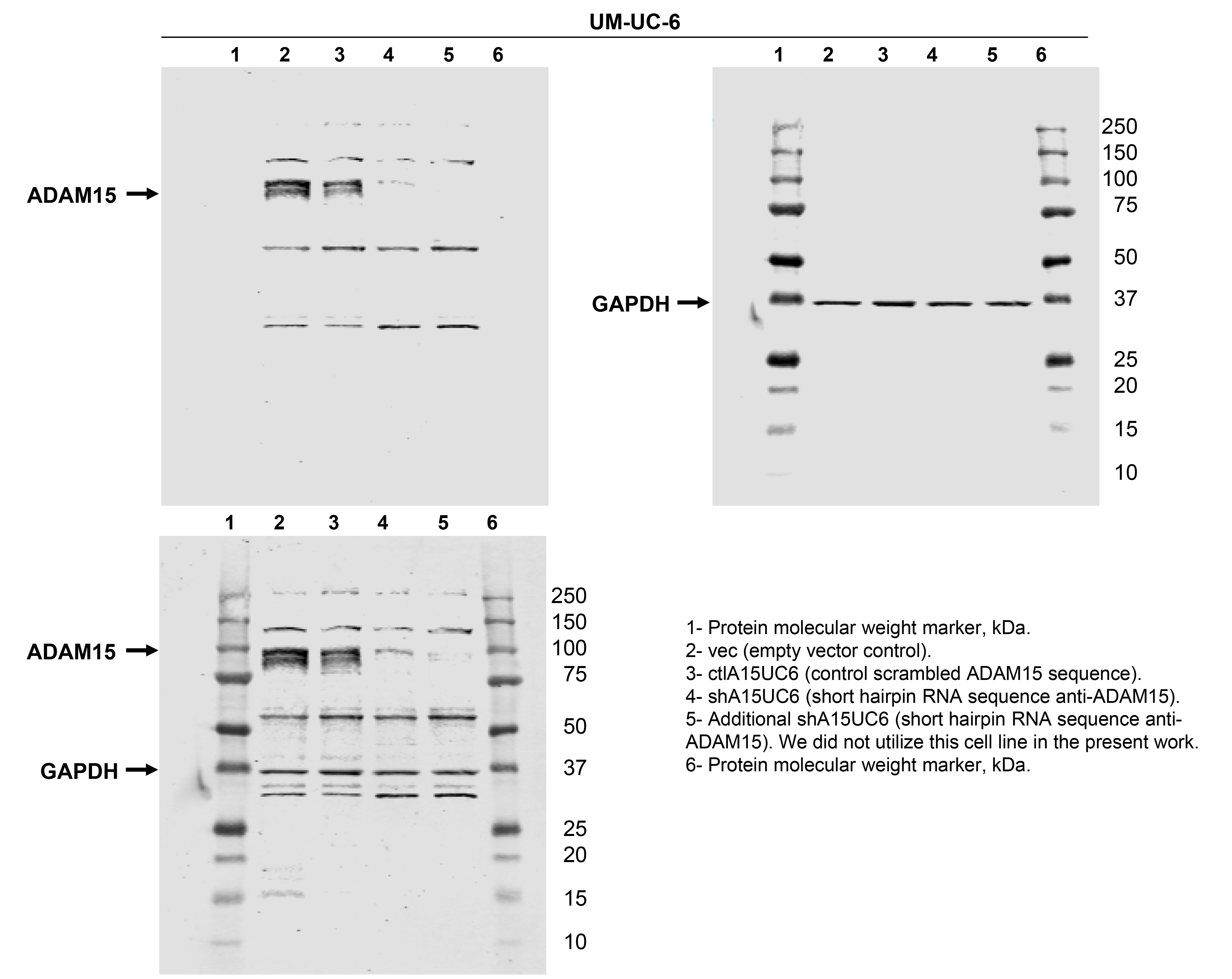

Supplement: S4 Fig — (TIFF) [file pone.0150138.s005.tiff]
