# Supplementary material for: ADAM15 Is Functionally Associated with the Metastatic Progression of Human Bladder Cancer
Source: PLoS One. 2016 Mar 1;11(3):e0150138. doi: 10.1371/journal.pone.0150138 (PMC4773041; doi:10.1371/journal.pone.0150138)
Supplement: S1 Table — (PDF) [file pone.0150138.s006.pdf]

Calculated Affinities in kcal/mol

	ADAM15	ADAM10	ADAM17	MMP9	MMP2
Adamastat	-9.0	-5.1	-8.1	-5.9	-7.9
PD166793	-6.4	-6.7	-8.6	-7.5	-7.8
Marimastat	-6.5	-7.2	-7.3	-6.5	-5.0

Affinities for the ‘best’ complex with ADAM15 catalytic domain active site of selected metalloproteinases
